# Essential Oil-Containing Polysaccharide-Based Edible Films and Coatings for Food Security Applications

**DOI:** 10.3390/polym13040575

**Published:** 2021-02-14

**Authors:** Arfat Anis, Kunal Pal, Saeed M. Al-Zahrani

**Affiliations:** 1 SABIC Polymer Research Center, Department of Chemical Engineering, King Saud University, Riyadh 11451, Saudi Arabia; szahrani@ksu.edu.sa; 2 Department of Biotechnology and Medical Engineering, National Institute of Technology, Rourkela 769008, India; kpal.nitrkl@gmail.com

**Keywords:** microbial food safety, polysaccharide, essential oils, edible films, edible coatings

## Abstract

The wastage of food products is a major challenge for the food industry. In this regard, the use of edible films and coatings have gained much attention due to their ability to prevent the spoilage of the food products during handling, transport, and storage. This has effectively helped in extending the shelf-life of the food products. Among the various polymers, polysaccharides have been explored to develop edible films and coatings in the last decade. Such polymeric systems have shown great promise in microbial food safety applications. The inclusion of essential oils (EOs) within the polysaccharide matrices has further improved the functional properties of the edible films and coatings. The current review will discuss the different types of polysaccharides, EOs, methods of preparing edible films and coatings, and the characterization methods for the EO-loaded polysaccharide films. The mechanism of the antimicrobial activity of the EOs has also been discussed in brief.

## 1. Introduction

Microbial food safety is a grave concern across the globe. This is because several microbes that include bacteria, viruses, and fungi are responsible for causing foodborne diseases. Foodborne diseases have been reported to significantly increase the healthcare cost of developing countries. As evident from the World Health Organization (WHO) report, published in the year 2015, nearly 600 million members of the population were affected by foodborne diseases in the year 2015, while more than 420,000 cases of mortality are being recorded each year across the globe [1]. The infection of such a large number of the population can be explained by the contamination of the food products by pathogenic microbes. The economies of most developing countries are dependent on the export of agricultural products. Hence, the microbial contamination of food products affects the economic growth of these countries, which further increases the economic burden. Keeping this factor in mind, various researchers are working towards the development of sustainable strategies for preventing the microbial contamination of food products. In other words, researchers across the globe have identified that enhancing the microbial safety of food products can drastically help developing countries in saving their economies. In this regard, the development of novel food packaging materials has evolved as a new area of research in food science in the last couple of decades.

Conventionally, food packaging materials (FPMs) are developed using plastics, which account for a sizable amount of petroleum oil consumption. It has been forecasted that by the year 2050, 20% of petroleum oil will be used for the manufacturing of plastics for different applications [2]. The use of plastics results in the overall carbon emissions of the earth, thereby negatively affecting nature. Hence, scientists are looking towards developing novel FPMs using biological sources that can allow reducing the use of plastics. The materials that are explored for the development of the FPMs include polymers from biological sources. Such materials are generally regarded as bioplastics since they are developed from natural sources. Many of the bioplastics are biodegradable in nature and do not pollute the environment [3]. The use of agrobiopolymers for the designing of biodegradable FPMs has received much attention in recent times. The agrobiopolymers are classified as polysaccharides, lipids, and proteins [4]. These polymers are extracted from agrowastes, which in turn further helps to reduce the environmental pollution burden. Many researchers have proposed various polysaccharide-based FPMs in recent times. Some of the commonly used polysaccharides include tamarind gum, chitosan, alginate, carrageenan, etc.

Further, it is important to note that FPMs have a significant role in food industries, starting with the collection of the raw food materials from the originating source (agricultural fields or animal farms) to processing, transportation, storage, and finally, consumption by the consumers. It is important to note that the food products provide a dynamic environment, where significant biological processes are ongoing. Hence, it is an extremely challenging task to design FPMs as these products are expected to retard or prevent the process of physiochemical degradation to keep the foods safe until consumption. In other words, modern FPMs are expected to considerably increase the shelf-life of the products. In this regard, food scientists are proposing the employment of active FPMs (AFPMs). The use of AFPMs can help control or prevent the microbial spoilage of the food products by preventing the growth of pathogenic foodborne microorganisms (e.g., *Salmonella*, *Listeria*) during the storage period, thereby not only enhancing the shelf-life of the food products but also keeping their quality and safety intact. Such AFPMs have entrapped antimicrobial compounds within their structures. The antimicrobial compounds can either be from natural or synthetic sources. Due to the instances related to the resistance to the synthetic antimicrobial compounds, the trend has shifted to the use of natural antimicrobial compounds. Some of the natural antimicrobial compounds include essential oils (EOs), organic acids, enzymes, antimicrobial peptides, and certain biopolymers (e.g., chitosan). The use of these natural antimicrobial agents for the development of the AFPMs has now become one of the most popular strategies across the globe. However, the use of the antimicrobial compounds in excess amounts may significantly affect the sensorial properties of the food products. Hence, the incorporation of these compounds within the AFPMs requires optimization that is achieved by extensive research.

Among the different antimicrobial compounds mentioned above, EOs (e.g., clove oil, lemon oil, cinnamon oil, tea tree oil, lavender oil, oregano oil, and peppermint oil) have evolved as one of the preferred natural antimicrobial compounds for the development of the AFPMs. The EOs are produced by the plants as the secondary metabolites, which are highly lipophilic, volatile, and prone to oxidative and photothermal degradation. Chemically, the EOs are composed of aldehydes, monoterpenes, flavonoids, isoflavones, carotenoids, alkaloids, and phenolic acids. The high lipophilic nature of the EOs makes them insoluble in water. Additionally, the EOs must be handled carefully due to their highly volatile natures. The main advantage of the EOs is their antibacterial activities against a wide spectrum of microbes. Since the EOs are extracted from natural sources, they are generally safe for human consumption. Due to this reason, many of the EOs have been categorized as generally recognized as safe (GRAS) by the Food and Drug Administration of the United States (US-FDA). The US-FDA has approved many EOs as an additive for food applications. The EOs are usually entrapped (encapsulated) within the polymeric films. The entrapment within the polymeric matrices extensively reduces the volatility of the Eos, thereby improving their efficiency. The polymeric architecture provides a platform that helps in controlling the release of the EOs from the films over the surface of the food in a sustained manner [5]. Additionally, the use of EOs for the development of the AFPMs significantly increases the contact area with the foods. This is accounted to the hydrophilic nature of the biopolymers. The biopolymers are inherently hydrophilic in nature. Hence, they will prevent the crossover of the hydrophobic EO components, thereby, helping in masking the odor of the EOs.

A large number of researchers have reported the use of EOs as active agents for the development of AFPMs. In the present review, a discussion on the polysaccharides that have been explored for the development of the EO-containing AFPMs will be made. Then, the mechanism of the antimicrobial activity of EOs, and different types of EOs that has been used for developed AFPMs will be discussed. Lastly, the synthesis and characterization methods of the polysaccharide and EO-based AFPMs will be discussed. 

## 2. Polysaccharides

Edible films and coatings consist of polymeric matrices that are developed with edible polymers (e.g., polysaccharides and proteins). The polymeric matrices are quite thin and are used for the packaging of food products. Said films and coatings are expected to prevent gaseous exchange, microbial spoilage, and loss of moisture and solutes from the food products without compromising their physicochemical and sensorial properties. Additionally, the polymer matrices can be consumed directly along with the packed food products. These polymeric matrices are prepared using edible biopolymers. Most of the biopolymers that are being explored are being obtained either from renewable sources or from the byproducts of the agroindustries. Since most of the biopolymers are obtained from natural sources, they are biodegradable in nature and hence are environment-friendly. The use of these biopolymers has shown significant promise to reduce the usage of nonbiodegradable plastic packaging materials. Further, as these polymers are obtained from natural sources, the food industries have a significant economic advantage. In recent years, the interest in polysaccharide-based edible films and coatings has seen a surge. This can be ascribed to the ease of chemical modification of the polysaccharides, which paves the way for the researchers to easily modulate the properties of the matrices of edible films and coatings. The polysaccharides are broadly categorized as starch and nonstarch polysaccharides. The nonstarch polysaccharides are more hydrophilic than the starch polysaccharides. The aqueous solutions and dispersions of long-chain hydrophilic polysaccharides help to enhance the viscosity of the solutions. The addition of these polysaccharides helps to tailor the hardness, crispness, and adhesiveness of the biopolymer matrices [6]. The nonstarch polysaccharides are categorized as cellulose and derivatives (e.g., carboxymethylcellulose, hydroxypropyl cellulose, methylcellulose, hydroxypropyl methylcellulose, microcrystalline cellulose, chitosan, gellan gum), seaweed extracts (e.g., carrageenans, alginates, agar, furcellaran, pectin), microbial fermentation gum (e.g., xanthan gum), exudate gums (e.g., gum arabic, gum tragacanth, gum karaya), and seed gums (e.g., tamarind gum, guar gum, locust bean gum) [6]. The current section will discuss in brief some of the polysaccharides that are being explored in recent times by food scientists to develop edible films and coatings. 

### 2.1. Carrageenan

**Figure 1:**
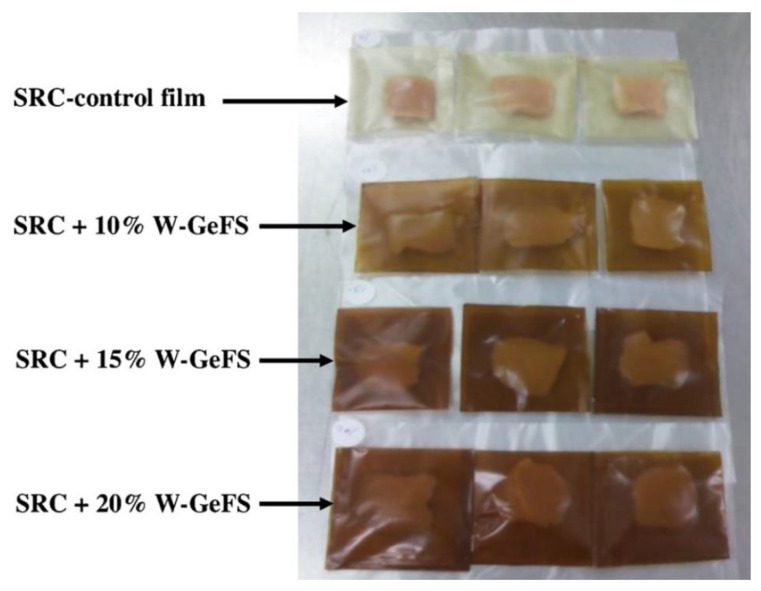
Photographs of chicken breast samples packed with the selected SRC films at 0 day. SRC: semirefined κ-carrageenan; W-GeFS: water extract of germinated fenugreek seeds. Reproduced with permission from [8]. Copyright 2020 Elsevier.

Carrageenan is a water-soluble linear-chain polysaccharide. Chemically, the biopolymer is regarded as the partially sulfated galactans. The polysaccharide is extracted from the cell walls of red seaweeds [7]. The biopolymer can be categorized either as kappa, iota, and lambda carrageenan, depending on their structural properties. Among the three categories of the carrageenan, kappa and iota types of carrageenan are commonly preferred as film-forming biopolymers. Since the carrageenans are hydrophilic in nature, they have been explored extensively as a film-forming agents. Polysaccharides consist of double helices, which undergo physical interactions to form a 3-D network when the carrageenan solutions are moderately dried. The 3-D network, so formed, induces the gelation of the polysaccharide matrices, which upon complete drying forms a stable film. Due to the film-forming ability of carrageenan, it has found extensive applications as edible films and coatings (Figure 1). The kappa-carrageenan can form films that have better strengths than the films developed from iota-carrageenan. This has been reasoned to the relatively lower hydrophilicity of the kappa-carrageenan as compared to the iota-carrageenan [8]. Unfortunately, refined kappa-carrageenans are very costly. Hence, in many cases, semirefined kappa-carrageenans are being used for the development of edible films and coatings.

### 2.2. Chitosan

**Figure 2:**
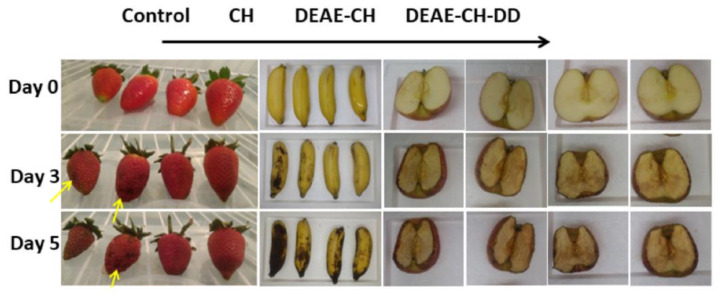
Visual decay of strawberries, bananas, and apples during storage time of 5 days. Each row shows the control on the left side, then the chitosan (CH)-, diethylaminoethyl chitosan derivative (DEAE-CH)-, and dodecyl grafted diethylaminoethyl chitosan derivative (DEAE-CH-DD)-coated antimicrobial edible films towards the right, respectively. The yellow arrows indicate signs of rot/spoilage. Reproduced with permission from [10]. Copyright 2020 Elsevier.

Chitosan is synthesized from the naturally occurring biopolymer chitin, by the process of N-deacetylation. Said biopolymer is an alkaline amino polysaccharide, which is the only biopolymer that occurs in nature [9]. The alkaline nature of chitosan can be proven by its solubility in mild acid solutions [10]. The polymer is regarded as the wonder biopolymer due to its inherent antioxidant and antimicrobial properties. This makes the biopolymer a choice of material as a preservative in food industries. Additionally, the biopolymer is well-known for its film-forming ability [11]. The film-forming capability of the films can be ascribed to the formation of the intermolecular interactions among the polymeric chains of the chitosan molecules [10]. Unfortunately, the chitosan-based films and polymeric architectures suffer from poor mechanical properties. This is the major limitation of exploring chitosan-based films for meaningful applications. To circumvent this problem, many researchers have used another component that can help improve the mechanical stability of the chitosan-based polymeric architectures. Some of the components include gelatin, starch, and alginates [9]. Further, different physical and chemical strategies of crosslinking of the chitosan matrices have been proposed to improve their mechanical properties. In recent years, the use of amphiphilic chitosans (e.g., diethylaminoethyl chitosans) has been proposed (Figure 2). This is because these amphiphilic chitosans have better solubility at neutral pH [10].

### 2.3. Alginate

**Figure 3:**
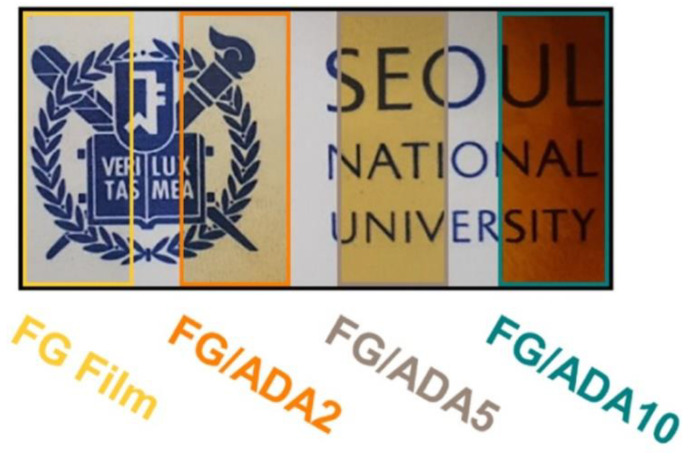
Optical properties of fish gelatin (FG)/alginate dialdehyde (ADA) composite films. Reproduced with permission from [13]. Copyright 2021 Elsevier.

The alginate biopolymer is extracted from brown algae and is biodegradable. Chemically, alginates are composed of (1→4)-linked-α-L-guluronate and (1→4)-linked-β-D-mannuronate moieties. The biopolymer is a linear anionic polysaccharide. The ionic nature of the alginates makes them highly soluble in water. Accordingly, they have been explored as film-forming biopolymers. The films that are made with alginates are transparent and of good quality. The films and coatings of alginates provide sufficient barrier properties to the external factors. Due to their excellent barrier properties, the alginate films and coatings hinder the permeation to oils, fats, moisture, and oxygen across it. The hindrance to the oxygen permeation prevents the oxidation of the food constituents, while the hindrance to the moisture loss prevents shrinkage of the food products [12]. Further, the films and coatings of alginates also provide excellent barrier properties to the microbes that help reduce the chances of microbial growth over the food materials. Additionally, the alginate-based films and coatings help to reduce the flavor, and color loss [12]. Partially oxidized alginates with free aldehydic groups have been used as a crosslinking agent for the protein (e.g., gelatin)-based edible films (Figure 3) [13]. The use of the partially oxidized alginates combines the advantages of aldehydic crosslinking without compromising the edible nature of the films, which is not the case for the synthetic aldehydes such as glutaraldehyde, formaldehyde, and glyceraldehyde [13].

### 2.4. Cellulose Derivatives

**Figure 4:**
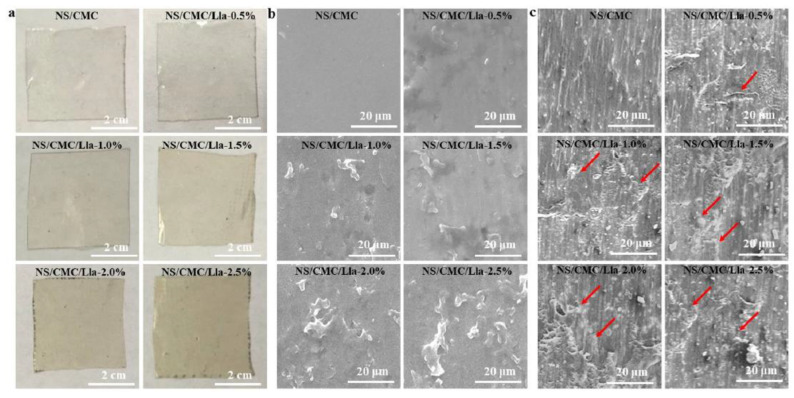
(**a**) Digital photos, (**b**) SEM images, and (**c**) cross-section images of NS (Corn Starch)/Carboxymethyl cellulose (CMC) with 0%, 0.5%, 1.0%, 1.5%, 2.0% and 2.5% *Lactococcus lactis*. Reproduced with permission from [15]. Copyright 2021 Elsevier.

Many of the cellulose derivatives are approved by the U.S. Food and Drug Administration (US-FDA) for food and pharmaceutical applications. In general, the cellulose derivatives are good film-forming agents, which is promoted by their hydrophilic nature. Hence, they have been explored extensively as edible films and coatings. They are inherently biocompatible (nontoxic), biodegradable, and nonimmunogenic in nature, which makes them suitable for human use/ consumption. Apart from the film-forming agents, cellulose derivatives are also used as emulsifiers, protective colloids, adhesives, and thickeners [14]. Carboxymethyl cellulose (CMC) is a semisynthetic anionic polysaccharide and can be easily synthesized at a low cost. The CMC is synthesized by the carboxymethylation of the cellulose backbone. Films formed of CMC are transparent and exhibit excellent mechanical properties, which are exceptionally beneficial (Figure 4) [15]. The addition of plasticizers further improves the properties of the CMC films, which are inherently brittle in the absence of the plasticizers [15]. Unfortunately, the films based on CMC alone have found limited application as edible films and coatings. This has been reasoned to the poor moisture barrier properties of the CMC-only films [16]. This has necessitated the development of CMC films in conjunction with other biopolymers. The films synthesized with the blends of CMC and other biopolymers (e.g., chitosan, starch, and collagen) were found to improve the shortcomings presented by the CMC-only films. Similar to CMC, cellulose acetate phthalate (CAP) is also a semisynthetic biopolymer that has greatly been explored in pharmaceutical industries as coating materials for enteric-coated tablets and capsules. The polymer is a crystalline polymer. Akin to CMC, CAP is also hydrophilic and biodegradable. The polymer was recently explored in conjunction with chitosan to develop nano-zinc oxide reinforced food packaging films [17]. Cellulose acetate (CA) is another semisynthetic cellulose derivative that has been explored for food packaging applications [18,19]. Some of the other cellulose derivatives that have been extensively explored as coating materials in both food and pharmaceutical industries include hydroxyethylcellulose (HEC) [20] and hydroxypropyl methylcellulose [21], in coating materials for enteric-coated tablets and capsules.

### 2.5. Agar

**Figure 5:**
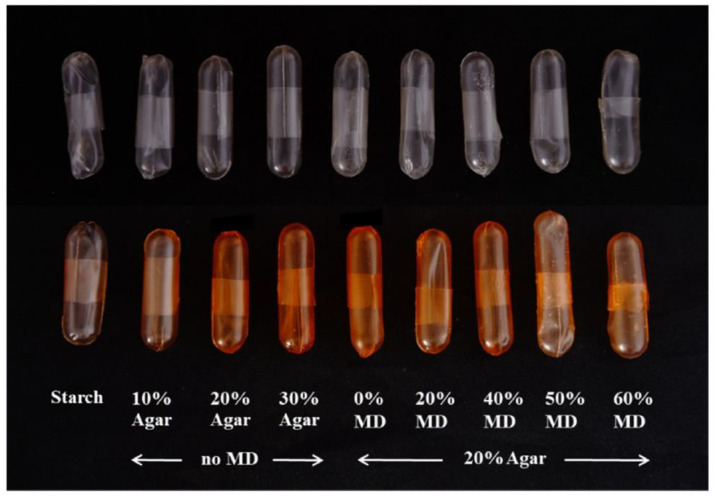
The appearance of capsules prepared by mold dipping with various ratios between starch, agar, and maltodextrin (MD). Reproduced with permission from [23]. Copyright 2020 Elsevier.

Agar is a marine biopolymer which is extracted from red algae. The biopolymer is categorized as Generally Regarded As Safe (GRAS) by the US FDA. It received the GRAS status in the year 1972 [22]. Since then, the biopolymer has been used extensively in food and biomedical applications (Figure 5). Nearly 80% of the agar that is produced globally is used for human consumption. The biopolymer forms good quality films. However, the films that are formed by the agar are highly brittle and possess poor moisture barrier properties and thermal stabilities [22]. A significant number of studies are being carried out to overcome the aforesaid disadvantages of agar films and to make the agar-based films suitable as edible films and coatings. Some of the strategies employed to improve the quality of the agar films include nanoparticle reinforcement and making blends of agar with biopolymers.

### 2.6. Tamarind Kernel Powder and Tamarind Starch and Polysaccharide

Tamarind (*Tamarindus indica* Linn; family: *Leguminosae*) seeds are the superfluous product of the tamarind-based agro and food industries. The tamarind seeds constitute ~30–40% of the whole fruit weight [24]. Tamarind kernel powder (TKP) is extracted from the tamarind seeds. The TKP has evolved as a promising raw material for food processing and food packaging applications in the last decade. Since TKP is extracted from unwanted products, the synthesis of edible products leads to the valorization of the industrial wastes, which helps in reducing carbon emission. The TKP, which appears as powder, contains a significant amount of proteins and polysaccharides. Due to the presence of proteins and polysaccharides in very high amounts, the TKP can form good quality films that have shown excellent properties to be used for food packaging applications. Akin to the previously mentioned polysaccharides, TKP is also biodegradable, biocompatible, and nontoxic. Similar to TKP, tamarind starch is also extracted from tamarind seeds. Tamarind starch has also been explored for developing edible films [25].

### 2.7. Pectin

**Figure 6:**
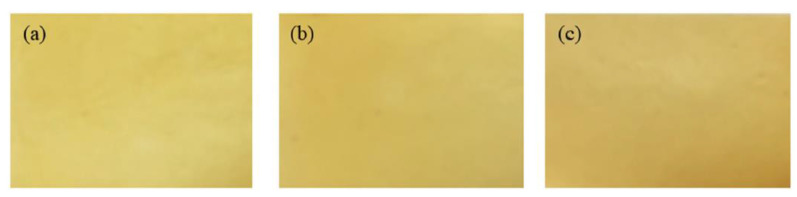
The appearance of Averrhoa bilimbi pectin (ABP)-based edible film (EF) extracted using choline chloride–citric acid monohydrate-based deep eutectic solvent (DES) at molar ratios of 3:1, 1:1 and 1:3—(**a**) EF-ABP3:1, (**b**) EF-ABP1:1, and (**c**) EF-ABP1:3. Reproduced with permission from [28]. Copyright 2020 Elsevier.

Pectin is a heteropolysaccharide and is extracted from plants and fruits. The biopolymer is the major constituent of all the plants and is responsible for the significant mass in the plants [26]. Most of the commercially available pectins are extracted from apple pomace and citrus peel [27]. It has emerged as a prominent biopolymer for food and biomedical applications due to its inherent biocompatible nature. The biopolymer is majorly constituted of linear homogalacturonans (smooth region), rhamnogalacturonan-II, and highly branched rhamnogalacturonan-I regions [27]. The homogalacturonans are present in the highest amount. The homogalacturonans are connected by α-D-galacturonic acid (GalA) by means of the 1→4 glyosidic bonds [27]. The carboxyl group of α-D-galacturonic acid may appear as a methylated ester. The extent of methylation can alter the properties of the pectins significantly. Based on the extent of methylation, pectins are categorized either as high methoxyl pectin (degree of methylation >50%) and low methoxyl pectin (degree of methylation < 50%) [27]. The structuring properties of the pectins are dependent on the extent of methylation. The structuring properties of the pectins have been well explored to tune the physical properties of the food products. The tuning of the physical properties is achieved by mixing pectins with different degrees of methylation. The edible films and coatings of pectins have good mechanical properties. Further, the mechanical properties of pectin-based edible films and coatings can be tailored by blending with proteins and polysaccharides (Figure 6) [26].

### 2.8. Soluble Soybean Polysaccharide

**Figure 7:**
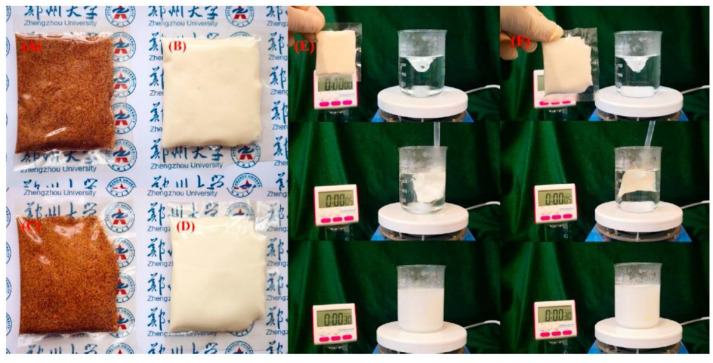
Heat sealable soluble soybean polysaccharide (SPSS)/gelatin blend edible films for food packaging applications. Film (**A**,**B**) with instant coffee powder and (**C**,**D**) with coconut powder are SSPS/gelatin blend film pouch with weight ratios of 100/0 (S100G0) and 60/40 (S60G40) respectively. (**E**) coconut powder in S100G0 pouch and (**F**) coconut powder in S60G40 pouch during the dissolution tests in hot water (95 ± 2 °C). Reproduced with permission from [30]. Copyright 2020 Elsevier.

The soluble soybean polysaccharide (SSPS) is an anionic polyelectrolyte and has a chemical structure that resembles pectin chemistry [29]. The biopolymer is extracted from the soy cotyledons [30] and is usually a byproduct of the soy protein production industry [29]. Chemically, an SSPS has a galacturonan backbone. The galacturonan backbone is composed of short-chain homogalacturonan and long-chain rhamnogalacturonan. Several branches within the galacturonan backbone consist of sugar chains of β-1,4-galactan and α-1,3 or α-1,5- arabinan chains [29,30,31]. The biopolymer is highly soluble in water, biodegradable, and biocompatible in nature. It also possesses viscosity building and antioxidant properties [29]. Further, these biopolymers have been explored as emulsifiers and film-forming agents in food research (Figure 7) [30]. This biopolymer has also been reported to have nutritional values [31]. The properties of the SSPS films have been tailored using plasticizers and essential oils [31]. Studies on tailoring the properties of SSPS films by nanoparticle reinforcement have also been conducted by many researchers [32,33].

### 2.9. Mushroom Polysaccharides

**Figure 8:**
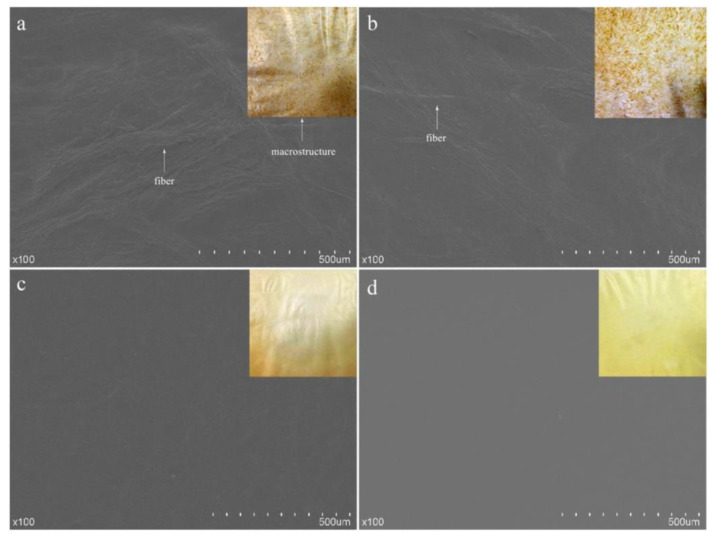
SEM images of surface morphology of film composed of different *Flammulina vellutipes* insoluble fibers prepared using (**a**) Colloid Mill (CM); (**b**) PFI Mill (PFI); (**c**) Low Pressure Homogenization (LPH); (**d**) High Pressure Homogenization (HPH), along with corresponding photographs. Reproduced with permission from [35]. Copyright 2018 Elsevier.

The mushroom polysaccharides are extracted from the fruiting bodies, sclerotia, and mycelium of oyster mushroom (*Pleurotus ostreatus*) [34]. The said mushroom is the most commonly consumed mushroom across the globe, and consists of a quantum of insoluble edible fiber [35]. The polysaccharide is synthesized as a metabolite by the oyster mushroom [34]. The polysaccharide mainly consists of β-glucan polymer units, which are linked to monosaccharide units through glycosidic linkages [36]. The polysaccharides have excellent nutritional properties. The polysaccharides have several beneficial biological properties, namely, antiaging, immunomodulatory, antioxidant, anti-inflammatory, anticancer, etc. [36]. Due to the natural origin of the mushroom polysaccharides, the polysaccharides are biodegradable in nature. Hence, they have been used to develop biodegradable films for food applications (Figure 8) [35]. Such films have been reported to have excellent oxygen and moisture barrier properties that are helpful in food packaging applications [35].

### 2.10. Konjac Glucomannan

**Figure 9:**
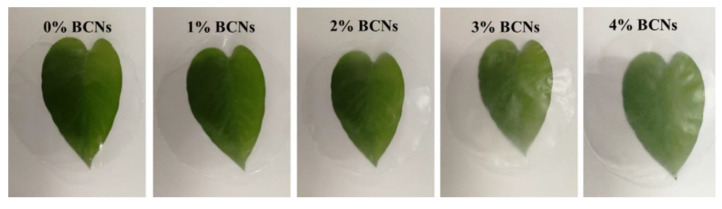
Light transmittance of konjac glucomannan (KG)-based edible films with different contents of bacterial cellulose nanofibers (BCNs) (0, 1%, 2%, 3% and 4%). Reproduced with permission from [37]. Copyright 2020 Elsevier.

The extraction of the polysaccharide konjac glucomannan (KG) is carried out from the tubers of the perennial herb konjac [37,38]. The polysaccharide is mainly composed of D-mannose and d-glucose monomers. The monomeric units are linked together by β-1,4-linking while the side chain is linked by β-1,6-glycosyl linking [37]. KG is a neutral polysaccharide that is soluble in water [38]. Akin to the other plant-derived polysaccharides, KG is a nontoxic, biodegradable, and viscosity modulator. It has been regarded as GRAS material by the US-FDA [38]. It also has an excellent film-forming property, which has been explored by food scientists to design edible films for food packaging applications. Unfortunately, KGM-only films suffer from poor mechanical and moisture barrier properties [35]. Hence, KG has been used in conjunction with other biopolymers (Figure 9) [35,39].

### 2.11. Chickpea Hull Polysaccharides

The polysaccharide extracted from the seed coat of chickpeas is chickpea hull polysaccharide (CHP) [40]. The quantum of the hull in the legumes is nearly 20%. The hull is composed of proteins and polysaccharides. CHP has excellent antioxidant properties. CHP has been blended with CMC to develop edible films [41].

### 2.12. Okra Mucilage Polysaccharide

Okra, also known as ladies’ fingers, is obtained from the plant *Abelmoschus esculentus* (L.) *Moech*. The fruit is highly nutritional and is rich in carbohydrates, proteins, minerals, and vitamins [42]. Okra is popular due to the presence of mucilage. The mucilage is rich in random coil polysaccharides. The okra mucilage polysaccharide (OMP) is composed of the sugars galactose, rhamnose, and galacturonic acid [42]. Pectin is the major component of OMP. Similar to other polysaccharides, OMP is nontoxic, biodegradable, and cheap. It also exhibits viscosity building properties. It has been used in conjunction with corn starch and carboxymethyl cellulose to develop edible films [42,43].

### 2.13. Cashew Gum

**Figure 10:**
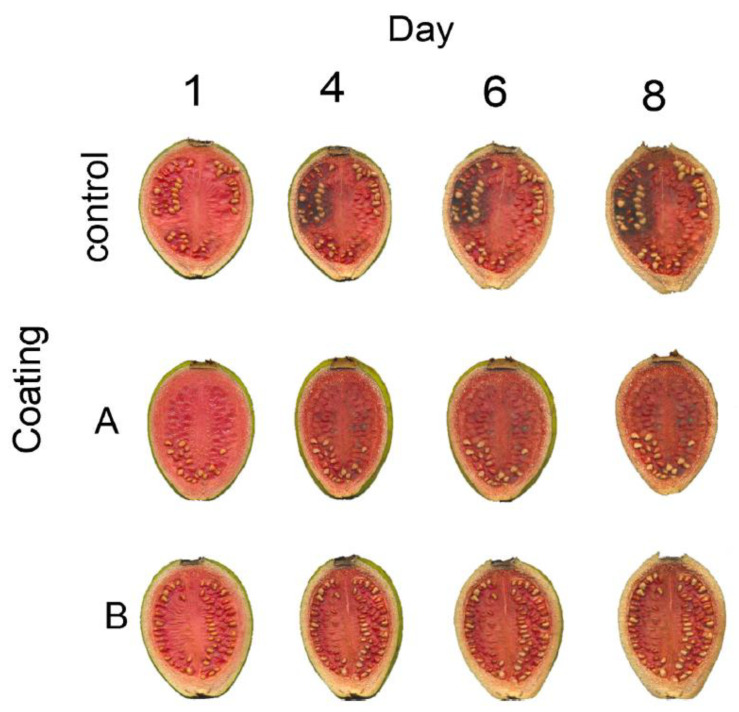
Photographs showing the visual aspects of guavas pericarp as storage at room temperature. The dehydration and change of color are evident for all samples. For control slices, the fungi proliferation is evident after the day 4. Reproduced with permission from [44]. Copyright 2015 Elsevier.

Many researchers have explored the application of cashew gum, a water-soluble high molecular weight anionic polysaccharide, for food applications [44]. The gum is categorized as a complex heteropolysaccharide, which is extracted from the exudate from the cashew tree (*Anacardium occidentale* L.) [45]. Chemically, the gum majorly consists of β-d-galactopyranose, α-d-glucopyranose, α-L-arabinofuranose, α-l-rhamnopyranose, and β-d-glacturonic acid [46]. The anionic nature of the gum has been accorded to the glucuronic acid [46]. The polysaccharide has shown a good promise for developing edible coatings due to its film-forming abilities (Figure 10). However, the use of polysaccharides has been recommended to develop polymeric architectures with ductile properties. The polymeric architectures of cashew gum exhibit antifungal and antibacterial properties. It has been reported that due to its inherent antimicrobial properties, the polymeric architectures of cashew gum can help prevent food from pathogenic and spoilage bacteria [44].

## 3. Essential Oils

The composition of the EOs is very complex. A typical EO would constitute more than 300 types of components. The EOs consist of 85–99% of volatile compounds [43]. The amount of each component is specific for the EO from a particular plant source. Further, the chemical constituent for a specific plant source can also vary depending on the geographical location of the collection. The alterations in the environmental conditions also affect the composition of the secondary metabolites. In essence, the composition of the EOs, in general, varies significantly. In general, the EOs consist of a variety of volatile chemicals that include terpenes, alcohols, ketones, phenols, aldehydes, amines, and amides [47]. These volatile compounds are low molecular weight components. Usually, the molecular weight of such components is approximately 300 Da. However, some of these components may have molecular weights of up to 1000 Da. Majorly, the bioactive components of the EOs are categorized as terpenes/terpenoids and aromatic/aliphatic compounds (Table 1). The quantum of terpenes/terpenoids in EOs is higher than the aromatic/aliphatic compounds. Some of the common terpenes/terpenoids include citronellol, geraniol, limonene, terpeniol, carvone, carvacrol, thymols, linalool, cineole, and camphor. These components usually form the major constituent of the EOs. Many EOs are constituted with 20–70% of the aforesaid components, which have been reasoned for most of the EO-associated biological activities [47]. The low molecular weight terpenes and terpenoids are the primary constituents responsible for the antimicrobial activity of the EOs. The composition of the EOs is analyzed mainly by chromatographic techniques such as gas chromatography, and ultra-high-performance liquid chromatography [43].

The EOs appear as colorless liquids. As mentioned earlier, these liquids are highly volatile, which can be ascribed to their aromatic natures. These volatile liquids are the secondary metabolites produced by the plants and hence are present in most parts of the plants—namely, seeds, flowers, peel, stem, bark, and whole plants [47]. They are reported to play an important role in the defense mechanism of the plants. This can be explained by the antimicrobial activity of the EOs. The antimicrobial activity of the EO was first reported De la Croix during the year 1881. The study reported the antimicrobial activity of the EO vapor. Thereafter, the EOs from different sources have been tested for different biological activities. The majority of the EOs from different sources have been reported to possess a wide range of biological activities that include antibacterial, antifungal, antiviral, and insecticidal activities [47]. Apart from their antimicrobial properties, EOs inherently possess characteristic aromas and flavors. Due to this reason, the EOs have found profound applications in food preservation. 

**Figure 11:**
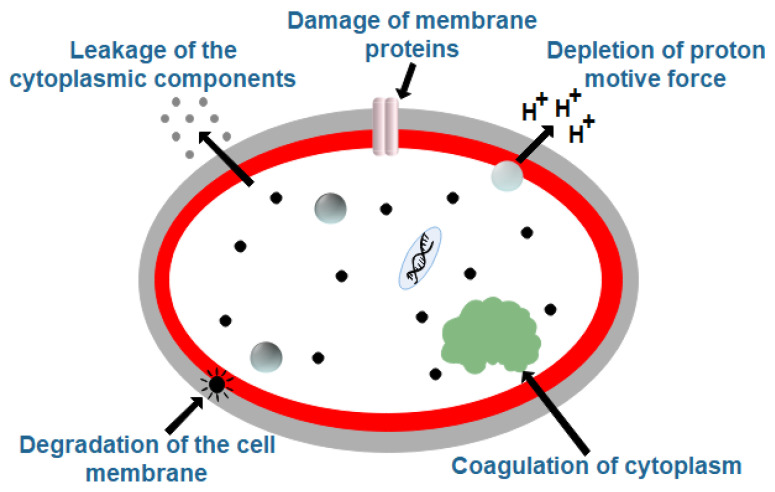
Plausible mechanisms of antimicrobial activity of the essential oils. Redrawn from [43].

Detailed information on the mechanisms of antimicrobial actions of EOs is not fully understood yet. However, various researchers have proposed several plausible mechanisms of antimicrobial activity (Figure 11). Most of the EOs increase the bacterial cell wall permeability. The increase in the permeability of the cell wall can be related to the dissolution of the lipid structures that compromises the integrity of the microbial cell wall [47]. This consequently results in the leakage of the intracellular contents, thereby resulting in bacterial cell death. In addition to the cell wall damage, the EOs can induce damage to the cell membrane proteins and cytoplasmic membrane. Hence, the EOs exhibit greater activity against the Gram-positive bacteria, which contains a peptidoglycan layer on the external surface of the outer membrane. The presence of the lipopolysaccharides in the Gram-negative bacteria prevents the internalization of the EOs within the bacterial cells. The activity of the quorum sensing response regulator of the genus *Vibrio* is inhibited by certain components of EOs (e.g., cinnamaldehyde and its derivatives), which considerably reduces their virulence [47]. Coagulation of the cytoplasm and reduction in proton motive force have also been reported in the presence of EOs. EOs that contain trans-cinnamaldehyde can significantly reduce the intracellular ATP levels in some bacteria (e.g., Escherichia coli and Salmonella Typhimurium) [47]. The reduction in the ATP levels can be accounted for by the combined effect of hydrolysis of the ATPs and hampering their synthesis. Interestingly, carvone can only disrupt the cell wall functionality and does not have any effect on the intracellular ATP levels in the bacteria. Many researchers have proposed the use of whole EOs for better antimicrobial activity as compared to the use of purified active antimicrobial components of EOs. This has been reasoned to the synergistic activity of the different antimicrobial components that are present in the EOs. The synergistic activity increases the potency of the EOs without inducing any toxic effects [47]. 

## 4. EO-Based Active Packaging Systems

**Figure 12:**
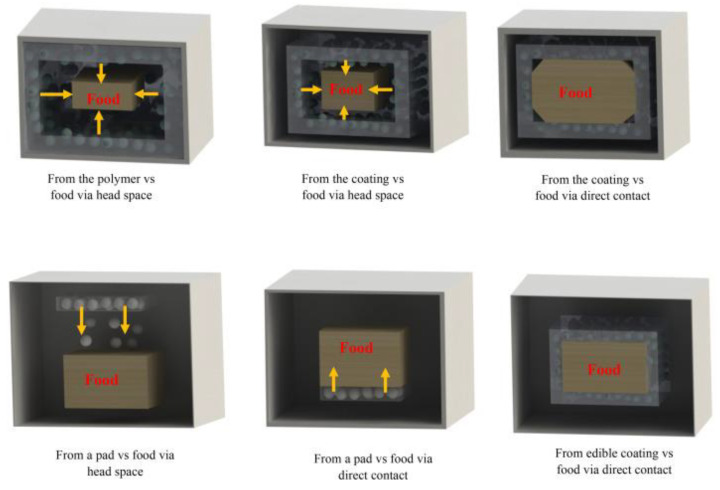
Possible methods for the release of antimicrobial agents in active packaging. Reproduced with permission from [1]. Copyright 2020 Elsevier.

**Figure 13:**
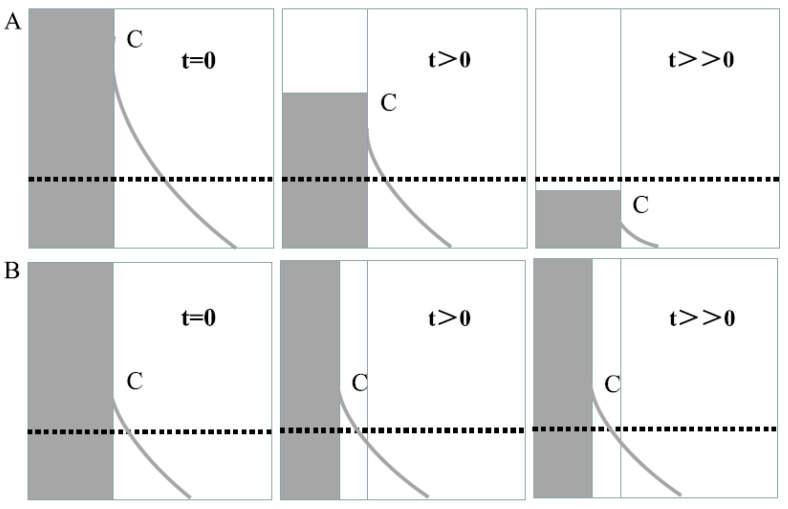
The migration curve of antimicrobial agents. (**A**) Release of soluble antimicrobial agents through free diffusion; (**B**): release of antimicrobial agents from monolithic system. Reproduced with permission from [5]. Copyright 2019 Elsevier.

As mentioned above, EOs are excellent antimicrobial agents and can be consumed orally without any untoward effects. Many of the EOs have been regarded as GRAS materials by the US-FDA. However, the EOs are volatile liquids, which makes them unsuitable for developing antimicrobial packaging systems. The EO-containing packaging (EO-AP) system consists of a polymeric film that houses EOs within the polymeric architecture. The polymeric films consist of several micropores. The micropores allow the migration of the EOs from the matrix phase to the surface of the films (Figure 12) [1]. Thereafter, it comes in contact with the food materials. In other words, EO-AP systems act as reservoir systems, and the EOs are released from the systems by the process of diffusion. It is important to note that if the EOs are miscible with the food materials, an unconstrained free diffusion of the EOs takes place within the matrix. This results in a decrease in the EO levels within the EO-AP systems as time progresses. After a certain period, the EO levels fall significantly, thereby resulting in an insufficient amount of EOs being released to the surface, which is lower than the minimum inhibitory concentration of the EOs. At this juncture, the EO-AP system fails to protect the food materials. On the contrary, in the case of EOs not being miscible with the food materials, the EO-AP systems act as monolithic systems from where a unilateral diffusion of EOs takes place. Herein, the EO content within the EO-AP systems essentially remains the same throughout the shelf-life of the food products. This results in the maintenance of the EO levels over the food surface at a predefined concentration. A mechanistic approach of the EO diffusion for both cases has been provided in Figure 13 [5]. As the concentration of the EOs over the food product surface is an important criterion that governs the shelf-life of the food products, the development of the EO-AP system with a suitable release profile is a must. Such a system is expected to allow the diffusion of the EOs in a controlled manner. In other words, the EO-AP system should act as a controlled delivery system for the EOs. The analysis of the kinetics of the diffusion of the EOs from the EO-AP system is an essential criterion. At any given point in time, the amount of EOs over the food surface should be more than the minimum inhibitory concentration (MIC) of the pathogens. Hence, the rate of diffusion of the EOs can affect the shelf-life of the food products. In general, an EO with a higher diffusion rate will quickly fall below the MIC levels, which would result in shorter shelf-lives of the food products. On the contrary, a sustained diffusion of EOs can significantly extend the shelf-life of the food products [5]. However, it has to be kept in mind that if the diffusion of EOs is too slow from the EO-AP system, this may result in a lower concentration of the EOs over the food surface. In this case, the EO-AP system will not be able to protect the foods at all. 

Further, it is important to note that apart from understanding the diffusion mechanics of the EO-AP composite film matrices, the insight on the growth kinetics of the microorganisms is also needed to be understood. This is because if the rate of microbial growth is higher than the rate of release of the EOs from the EO-AP system, then the quantum of the EO released would not be sufficient to control the growth of the proliferating microbes. It would lead to the spoilage of the food products quickly. This can be explained by the fact that the minimum inhibitory concentration (MIC) of the EO for inhibiting the growth of the microbes would not be maintained. On the contrary, if the release is too fast, the EO would be consumed quickly and that would also reduce the shelf-life of the food products. In this case, the amount of EO will be much greater than the MIC during the initial phase. However, as time would progress, the EO will be depleted from the reservoirs of the EO-AP matrix systems. This would ultimately result in the availability of the EOs in levels lower than the MIC.

### 4.1. Synthesis Methods

**Figure 14:**
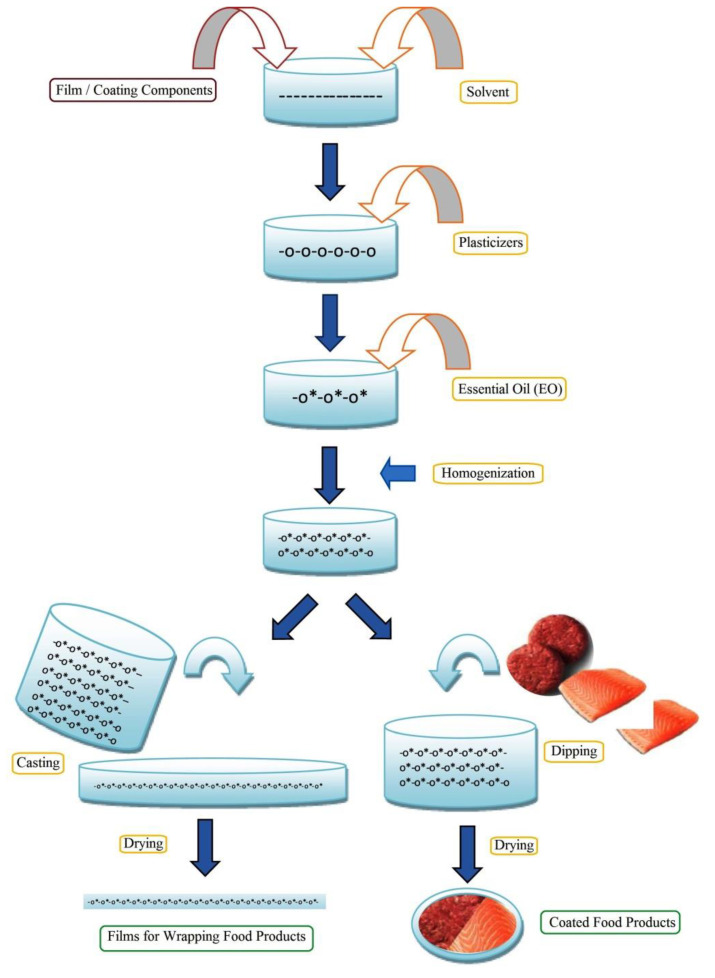
Preparation of polysaccharide-based edible film with tailored properties for food preservation. Reproduced with minor modification from [60]. Copyright 2020 Elsevier.

**Figure 15:**
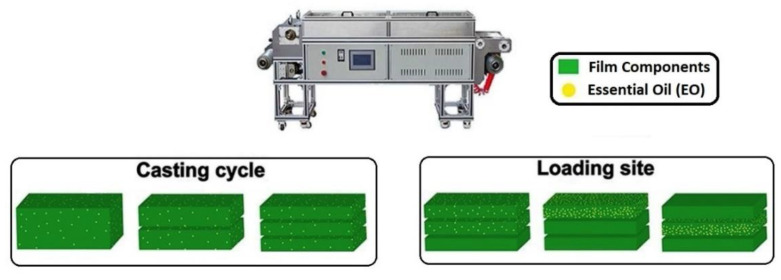
Schematic diagram showing casted films with different casting cycles and loading positions of Eos. Reproduced with minor modification from [59]. Copyright 2020 Elsevier.

The EO-APs are commonly developed by solution casting method. Initially, the film forming solutions (FFSs) are prepared by dissolving the polysaccharides (1–5 *w*/*v*%) in a suitable solvent such as deionized water, ethanol etc. The dissolution process may require stirring and heating to facilitate the dissolution of the polysaccharides. EOs are then generally incorporated in the FFS, and subsequently homogenized either by high-speed blenders or ultrasonication so as to form EO-in-polysaccharide solution emulsions, which are known as film-forming emulsions (FFEs). Placticizers (e.g., glycerol, polyethylene glycol) are often added to FFEs. The addition of the plasticizers helps to improve the flexibility to the films. The FFEs are then poured into molds, kept on a levelled surface, and then dried either at room or elevated temperature (Figure 14). As discussed previously, the EOs are highly volatile and have low water solubility. Hence, artificial surfactants (e.g., Tween 20, Tween 80, Lecithin, etc.) are normally utilized to prepare the emulsions of EOs. In recent years, edible proteins and polysaccharides have been explored to prepare pickering emulsions. Pickering emulsions ensure sustained release of the EOs. In this regard, zein protein (an FDA approved material for food applications) particles have been used to stabilize pickering emulsions [9,57,58]. The continuous casting machines are used for developing films on a large scale. The thickness of the films is controlled herein by adjusting the scraper height. The drying of the films is achieved within the drying chamber of the casting unit. Multilayered films can also be prepared by running multiple casting cycles. For the purpose, the FFEs are added over the previously casted layers (Figure 15) [59].

Alternatively, the FFEs are allowed to form a polymeric coating on the food product itself. This is performed by dipping the food products within the FFEs and drying them under controlled environmental conditions. The properties of these films can be further improved by physical or chemical crosslinking [24,29,60,61,62,63]. The coating of the films can also be achieved by the spraying technique, in which the food products are sprayed with the FFEs through a nozzle. The spraying is generally carried out inside a reactor or an enclosure where the environment temperature is precisely controlled. During the process, the food products are rotated to ensure uniform coating [64].

The EO-loaded films can also be produced by film extrusion method. However, the method is used for developing films using resins. The process can be used to produce single or multilayer films depending on the type of the extruder used. The produce films have been used to make bags or liner or covers which can preserve foods [65]. Since the current review is on the EO- and polysaccharide-based edible films and coatings, this method is out of the scope of the review.

Table 2 summarizes a selected group of compositions of the EOs and polysaccharides that have been explored in recent studies for making edible films and coatings. 

### 4.2. Characterization Methods

The interactions between the EOs and the polymers play an important role in not only governing the properties of the EO-APs, but also in the shelf-life and the quality of the food products. The nature of the migration of the EOs within the polymer matrices can govern the shelf-life of the food products. The physical properties of the EO-Aps, such as the microbial, gas exchange, and moisture barriers, are significantly governed by the nature of the interactions between the EOs and the polymer matrix. Further, the mechanical and the viscoelastic properties of the films are also dependent on the EO–polymer matrix interactions. An alteration in the EO content can result in a variation in the thickness and other aforesaid properties. The colors of the edible films and coatings vary with the nature and composition of the EO-AP composite system. Hence, in this section, we will discuss the different types of characterization techniques that are used for the analysis of the EO-APs.

#### 4.2.1. Thickness of the Films

The physical (e.g., barrier, water vapor transmission rate, moisture content, etc.) and mechanical properties of the films are influenced by the thicknesses of the films. Hence, the thickness of the EO-APs is generally measured. The measurement of the films is usually measured using a micrometer. The use of a micrometer having a least count of at least 0.001 mm has been proposed by the researchers [24]. It has been observed that the addition of EOs within the polymer matrices increases the thickness of the EO-AP films—e.g., the addition of geraniol increased the thickness of the tamarind kernel powder/geraniol films from 0.240 ± 0.03 (0.5% *w*/*w*) to 0.290 ± 0.06 mm (3.0% *w*/*w*) [24].

#### 4.2.2. Moisture Content Analysis

The determination of the moisture content within the polymeric films was carried out by the thermogravimetric analysis method. Simplistically, the films were incubated in a hot air oven that is maintained at 105–110 °C. The incubation of the films was continued until a constant weight of the films was obtained [95]. The weight of the polymeric films was recorded before and after the incubation. Herein, the films were heated by the process of convection. The moisture content (MC) within the films was calculated using Equation (1) [24].

(1) $$\mathrm{MC}\left(\%\right)=\frac{m_{1}-m_{2}}{m_{1}}\times 100$$

where *m*_1_ and *m*_2_ are the initial and final weights of the films, respectively.

Recently, the MC analysis of the films has been carried out using moisture analyzers. Moisture analyzers automate the process mentioned above. The thermogravimetric moisture analyzers consist of an infrared radiation source (mostly halogen lamps), which radiates infrared radiation and consequently increases the temperature within the moisture analysis chamber. The infrared radiations are absorbed by the samples, thereby increasing the sample temperature. The chamber is fitted with a weighing balance. The weighing balance helps to continuously monitor the change in the weight of the samples. The processor of the instrument displays the change in the weight of the samples in grams/milligrams or percentage moisture loss. The main advantage of the moisture analyzers is that the measurement can be carried out mostly within 5–15 min. It is reported that the addition of EOs reduces the moisture content of the films. This has been attributed to the reduction in the hydrophilicity of the films with the incorporation of the EOs, which in turn affects the interaction of the water molecules with the polymer chains [95,96]. A monotonous reduction in the moisture content with the increase in the geraniol (a type of EO) content within the tamarind kernel polysaccharide films was observed [24]. However, the determination of the moisture content of the EO-APs by the thermogravimetric method, both manual and automated, is quite debatable. This is because the EOs are volatile in nature and they also tend to evaporate within the experimental conditions.

#### 4.2.3. Water Solubility

The water solubility of the EO-APs is an important measure for the packaging materials, especially for the packaging materials that are meant to be in contact with high-moisture food products. It is expected that the packaging materials should withhold their integrity even in high-moisture environments. The hydrophilicity/hydrophobicity of the films can significantly alter the water solubility of the films. The water solubility of the EO-APs can be determined by putting an accurately weighed piece of the EO-APs into the deionized water, kept on agitation. The use of deionized water is preferred as the presence of ions in the water may alter the solubilization process. Further, the agitation of the water is recommended during the solubilization process. The agitation helps to prevent the formation of the saturation layer around the films that may hinder the solubilization process of the films. The addition of EOs is reported to increase the lipophilicity of the EO-AP films. It has been observed that, due to this reason, the addition of EOs lowers the water solubility of the EO-AP films as compared to the pristine polymeric films [97]. Among the EO-containing films, films with higher EO contents usually demonstrate lower water solubility. For example, the addition of geraniol increased the thickness of the tamarind kernel powder/geraniol films from 0.240 ± 0.03 (0.5% *w*/*w*) to 0.290 ± 0.06 mm (3.0% *w*/*w*) [24].

#### 4.2.4. Optical Properties

**Figure 16:**
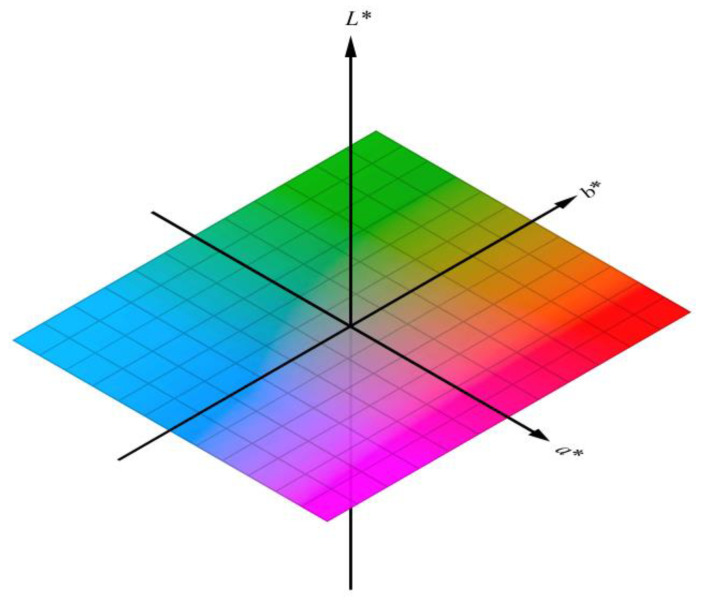
CIELAB color space. Reproduced from [98] under Creative Commons Attribution-Share Alike 4.0 International license.

The visual appearance of the packaging material plays an important role in consumer acceptability. Hence, the optical properties of the packaging materials were analyzed in-depth by measuring the color parameters of the films. The colorimetric analysis of the films was carried out in the reflection mode for the opaque films, and in the transmittance mode for the films that contain a certain degree of transmittance [1]. The widely acceptable color model for the analysis of the food products including the food packaging materials is CIELAB color space. The CIELAB color space was defined by the International Commission on Illumination in the year 1976. It measures the color parameters in terms of L*, a*, and b*. The CIELAB color space has been depicted in Figure 16. The L* value is regarded as the lightness of the samples, and it varies from 0 to 100. Herein, 0 stands for black, and 100 stands for white. The a* value represents the variation in color that may vary from green to red. The greenish colors are represented as –ve a* values, while the reddish colors are represented as +ve a* values. Lastly, the b* values represent the variation in the color that may vary from blue to yellow. The blueish colors have –ve b* values, while the yellowish colors have +ve b* values. The combination of a* and b* values results in the generation of the hue, which is associated with the true color of the samples. The color parameters of the films are dependent on the color of the raw materials. The lightness and the whiteness index of the polymeric films increases as EOs are incorporated within the polymeric matrices. This has been ascribed to the dispersion of the light from the interface of the polymer–EO interface [97].

#### 4.2.5. Film Microstructure

**Figure 17:**
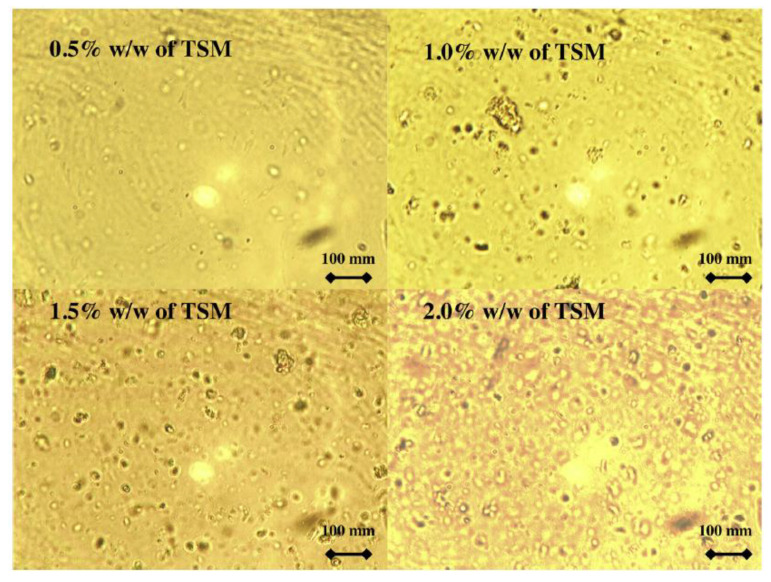
Optical microscopy images for 0.5%, 1.0%, 1.5%, and 2.0% (*w*/*w*) tamarind seed mucilage dispersions. Reproduced with permission from [104]. Copyright 2018 Elsevier.

**Figure 18:**
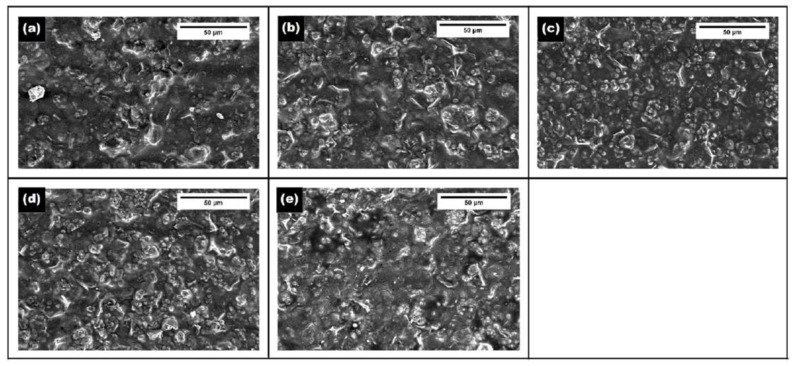
Environmental scanning electron microscopic images of tamarind gum film and geraniol (GER) containing films. Films with different GER content (w/w) (**a**) 0 %, (**b**) 0.5 %, (**c**) 1.0 %, (**d**) 2.0 % and (**e**) 3.0 %. Reproduced with permission from [24]. Copyright 2020 Elsevier.

**Figure 19:**
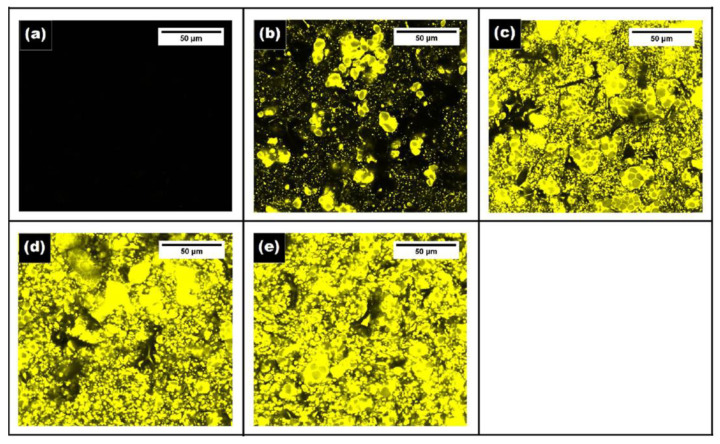
Confocal laser scanning microscopic images of tamarind gum film and geraniol (GER) containing films. Films with different GER content (w/w) (**a**) 0 %, (**b**) 0.5 %, (**c**) 1.0 %, (**d**) 2.0 % and (**e**) 3.0 %. Reproduced with permission from [24]. Copyright 2020 Elsevier.

The analysis of the microstructure of the films may divulge interesting information on the properties of the films. Hence, the microstructure of the EO-AP was analyzed. The use of a suitable microscopic technique may allow researchers to identify the arrangement of the different phases of the films [99]. The arrangement of the different phases has been reported to alter not only the optical properties but also the mechanical and barrier properties of the EO-AP films [24]. The microstructural arrangements within the films are dependent on the interfacial properties among the different phases. This, in turn, tailors the dispersion characteristics and the EO droplet morphology within the biopolymeric films [100]. The process parameters employed during the preparation of the films can also alter the film characteristics. This is because the use of suitable process parameters can prevent emulsion destabilization (i.e., flocculation, coalescence, and creaming of the EO phase) during the evaporation phase of the film formation [100,101]. The microstructural analysis can be carried out by different microscopic methods—namely, bright-field microscopy (Figure 17), scanning electron microscopy (Figure 18), and confocal microscopy (Figure 19). The biopolymeric films are relatively smooth. Incorporation of the EOs within the biopolymeric matrices increases their roughness significantly. In general, an increase in the EO content increases the roughness that results in the formation of a heterogenous surface structure [24]. The distribution of the EOs within the biopolymeric matrices can be analyzed by confocal laser scanning microscopy (CLSM). The analysis technique involves the solubilization of a chromophore (e.g., Fluorol Yellow 088). The distribution of the chromophore within the biopolymeric matrices is then visualized by CLSM [102,103].

#### 4.2.6. Water Vapor Transmission Rate (WVTR)

The transmission of water vapor molecules plays an important role in food packaging and coatings. The food packaging and coatings are expected to retain moisture within the food products by retarding the loss of moisture across the developed films. The films that can retard the moisture migration to the external environment have been found to increase the shelf-life of the food products [61,105]. The WVTR of the films was estimated by gravimetric analysis. The films were used to seal the glass vials that contain water. The vials were then transferred to a thermal cabinet that was maintained at 25 °C and 45% RH. The vials were initially allowed to equilibrate for 1 h. Thereafter, the weights of the vials were recorded for a particular duration at regular intervals. Then, the loss of the moisture was then plotted against time. The WVTR values in g/h·m^2^ were then estimated from the plot [61]. The addition of EOs within the polysaccharide-based films showed a significant decrease in the WVTR. The reduction in the WVTR was explained by the ability of the EOs to tailor the tortuosity factor [96].

#### 4.2.7. Fourier-Transform Infrared Spectroscopy (FTIR)

**Figure 20:**
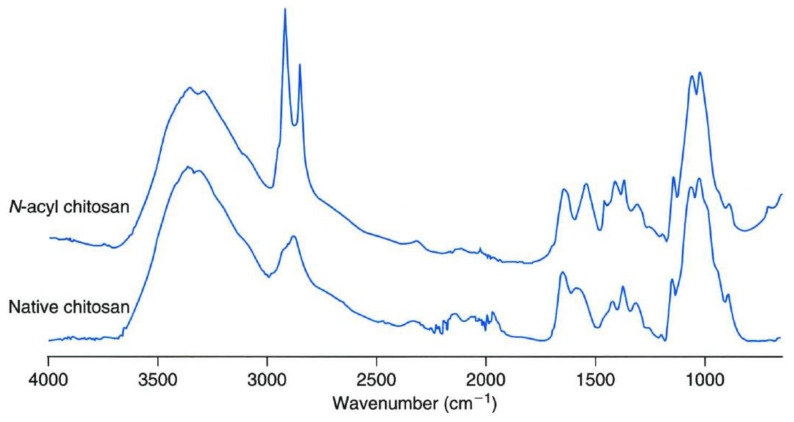
Fourier-Transform Infrared Spectroscopy (FTIR) spectrogram of the polysaccharide films. Reproduced with permission from [6] Copyright 2005 Elsevier.

FTIR analysis of the films was generally carried out in attenuated total reflectance (ATR) mode. ATR-FTIR analysis is a nondestructive way to analyze the film samples. For the analysis, the films were placed in contact with the ATR crystal, which is made up of either diamond in a high-valued instrument and zinc selenide in cheaper spectroscopes. It was ensured that the films form firm contacts with the ATR crystal. Biopolymeric films usually show an absorption peak of the hydroxyl group stretching at a wavenumber of ~3350 cm^−1^ (Figure 20). The C-O stretching vibrations of the monosaccharides that are present in the polysaccharides were observed at ~1050 cm^−1^. Agarwal et al. (2020) have reported that the addition of geraniol results in a significant alteration in the broad peak which is present in the wavenumber region of 3700−2900 cm^−1^. The authors concluded that the addition of EOs alters the hydrogen bonding within the biopolymeric matrices. It was observed that at the lowest geraniol content, the hydrogen bonding was increased. However, an increase in the geraniol content monotonously reduced the hydrogen bonding [24].

#### 4.2.8. X-ray Diffraction (XRD) Analysis

**Figure 21:**
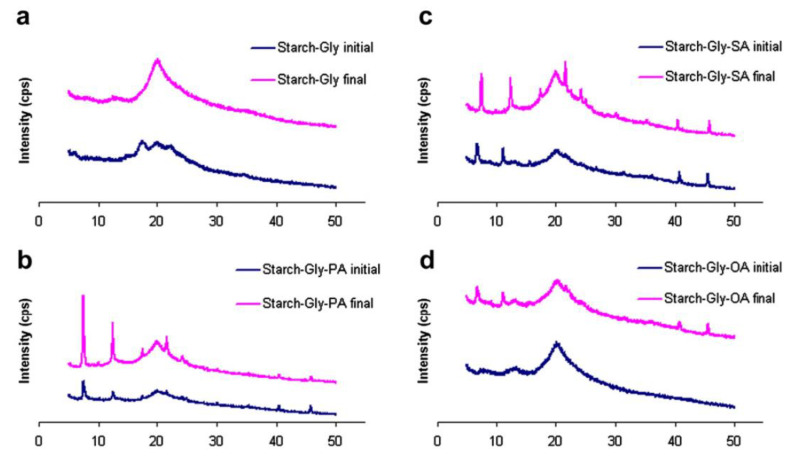
X-ray Diffraction (XRD) profiles of glycerol (Gly) plasticized starch films (non-stored and stored films for 5 weeks) with different fatty acids (**a**) Blank, (**b**) Palmitic acid (PA), (**c**) Stearic acid (SA), and (**d**) Oleic acid (OA). Reproduced with permission from [100]. Copyright 2012 Elsevier.

The XRD analysis allows the analysis of the molecular interactions within the samples in a nondestructive manner. The analysis allowed us to estimate the d-spacing (interplanar spacing), crystallite size, and crystal defects within the samples. The polysaccharide-based films usually showed a characteristic broad peak at ~20° 2θ (Figure 21) [106]. The same was observed in the polysaccharide-based EO-AP films [24]. In general, an increase in the EO content can increase the d-spacing with a concurrent decrease in the crystallite size and increase in the lattice strain. Interestingly, at lower concentrations of EO, a decrease in the d-spacing and lattice strain, and an increase in the crystallite strain was also observed [24]. The variations in these parameters can be ascribed to the alteration in the hydrogen bonding within the films [107].

#### 4.2.9. Mechanical Test

**Figure 22:**
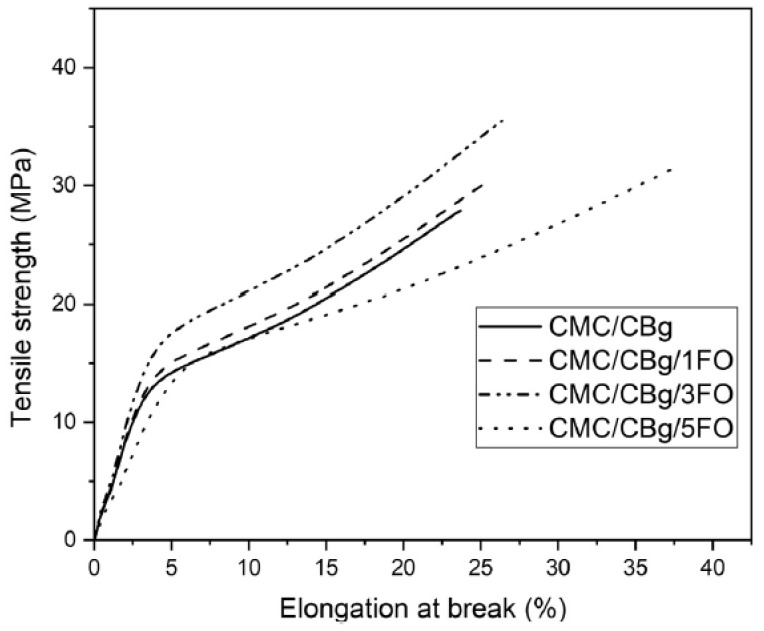
Tensile strength vs. elongation at break for carboxymethyl cellulose (CMC)/chitosan biguanidine hydrochloride (CBg) and CMC/CBg/ frankincense oil (FO) films. Reproduced with permission from [16]. Copyright 2019 Elsevier.

The analysis of the mechanical properties of the edible films and coatings provides information on the probable durability of the films. Additionally, the mechanical properties can divulge the ability of the films and coatings to protect the food products from production to consumption. Various mechanical properties such as tensile strength, Young’s modulus, and percentage stress relaxation were determined to compute the efficacy of the edible films and coatings [24,108]. These properties were related to the microstructure of the films and coatings, and hydrogen bonding within the components of the films and coatings. The tensile strength at the breakpoint and Young’s modulus are determined by analyzing the films in the tensile mode. The film samples were extended at a speed slower than 1 mm/s. Some authors employed a higher speed of extension. However, a higher speed of extension is often discouraged. This is because, at higher speeds, the impact of stress can affect the outcome of the result. The tensile strength at the breakpoint of the biopolymeric films is generally in the range of 20–30 MPa (Figure 22) [24,109]. The addition of the EOs within the biopolymeric matrices results in the disruption of the network structure, which can reduce the tensile strength to as low as 10 MPa [24,110]. However, in some cases, an increase in the tensile strength of the films has been reported [96]. The wide variation in the tensile strength properties of the EO-containing films can be ascribed to the multifactorial variations including EO droplet size and distribution, the nature of the biopolymer(s) used, and the interaction between the EO and the biopolymer(s). The Young’s moduli of the films is related to its stiffness/rigidity. Inclusion of the EOs within the biopolymeric matrices usually reduces the Young’s moduli of the films. This is ascribed to the injunction of the structural deformity within the biopolymeric matrices, which consequently reduces the fracture strength of the films [111]. The stress relaxation studies provide a measure of the viscoelastic properties of the biopolymeric films. This study was also carried out in the extension mode. The inclusion of the EOs within the biopolymeric matrices has been reported to increase the fluidic nature of the films with a consequent decrease in the stored energy at the end of the relaxation process [24].

#### 4.2.10. Antimicrobial Test

The EOs generally exhibit antimicrobial activities due to the factors mentioned above. The inclusion of the EOs within the films was intentionally carried out to exploit the antimicrobial efficacy of the EOs. The edible films and coatings were expected to release EOs over the food products in a sustained manner such that the films and the coatings can hinder the process of microbial growth. The antimicrobial activities of the EO-APs is generally checked by disc diffusion assay [112]. In this study, the suspensions of the test microbes (e.g., *Bacillus cereus*, *Escherichia coli*, etc.) were spread over the culture plates of sterilized solidified media (e.g., nutrient agar, BHI agar media). Thereafter, films cut into circular pieces were placed over the solidified media. Then, the culture plates were incubated to allow the growth of the microbes. The antimicrobial activities of the films were analyzed by measuring the zone of inhibition around the pieces of the films. The antimicrobial activity of the films was dependent on the composition of the films. The edible coatings on food products were peeled off and then tested for antimicrobial activity in a similar manner to that mentioned for films. The nature of the biopolymer matrices plays an important role in the release profile of the EOs and can significantly affect the antimicrobial activity of the EO-APs [71]. The presence of the amino groups in the chitosan molecules imparts inherent antimicrobial properties on it. The incorporation of the EOs within the chitosan matrix synergistically increases the antimicrobial properties of the resultant films [108].

## 5. New Trends and Technologies

Currently, there is a considerable challenge to researchers to develop products with good customer acceptability. The sale of food products is now greatly regulated by consumer demands. Herein, the packaging of food products plays an important role. Unlike in the recent past, the packaging materials are expected to be of high quality. The industries are looking towards the use of sustainable natural materials such as proteins, polysaccharides, and essential oils to design safer food packages. The uses of nonbiodegradable and nonrenewable petroleum-based polymers are nowadays out of favor to design food packages. This has been attributed to the harmful environmental effects exerted by these polymers. The use of sustainable natural materials allows the researchers to design films and coatings which are edible unlike the packages made from petroleum-based polymers that are nonedible. The edible films and coatings are safe for human consumption. These films and coatings can not only help in extending the shelf-life of the food products by controlling the respiration/moisture evaporation process, but also help in preventing the growth of the pathogenic microbes. Currently, many of the edible films and coatings are as efficient as those of the modified-atmosphere packages.

Edible polymers are readily available in nature, and are therefore sustainable. Further, these polymers are usually biodegradable and biocompatible/nontoxic. However, a variety of polymers are available in nature. Each of these polymers has its unique beneficial properties. Some may have certain disadvantages too. For example, the films based on CMC are inherently brittle. In general, blends of edible polymers are used to develop the main matrix to achieve desirable properties for packaging applications, which differs for different types of food products. Hence, the major challenge for researchers is to identify a suitable combination of edible polymers and/or additives such as plasticizers that can fulfill the requirements for the packaging of the food products. Further, most of the techniques to develop such products employ wet methods, which makes the upscaling of these products by the industries quite cumbersome. It is expected that in the near future researchers will be able to overcome these challenges to develop suitable technologies that can help industries to upscale the synthesis of edible films and coatings of food products.

## 6. Conclusions

In the last decade, there has been an increased interest in the development of polysaccharide-based edible films and coatings for the protection of food products. These have been ascribed to the availability of the polysaccharides in nature, and hence are available at a low cost. Additionally, the polysaccharides are inherently biocompatible and biodegradable. These properties allow for the use of polysaccharides as edible films and coatings. The polysaccharide-based systems have shown great promise in extending the shelf-life of food products, which can be ascribed to the barrier effects exerted by such systems. Such systems have been used for the protection of a variety of food products that include vegetables, fruits, meat and meat products, seafood, and confectionery products. Their ability to extend shelf-life is mainly associated with their ability to prevent dehydration, rancidity due to oxidation, and enzymatic browning. Due to the barrier properties exerted by the edible films and coatings of polysaccharides, it is possible to control the internal atmosphere between the polysaccharide layer and the food products. The inclusion of the EOs within the polysaccharide matrices increases not only their antimicrobial activities, but also the antioxidant properties. The polysaccharide matrix has been found to tailor the release of the EOs, thereby sustaining the release of the EOs and the shelf-life of the food products. With the advancement in the field of polymer technology, it is expected that the quality of the polysaccharide-based edible films and coatings can be improved significantly in the years to come. 

## Figures and Tables

**Table 1:** Source and major components of some of the common essential oils [43].

Essential Oil	Source Plant/Herb	Part of the Plant	Major Components	References
Lavender essential oil	*Lavandula officinalis*, L. *angustifolia*, L. *latifolia*	Flowers	Linalool, linalyl acetate, lavandulol, geraniol, and bornyl acetate	[48]
Basil essential oil	*Ocimum Lamiifolium*	Leaves	Methyl chavicol, linalool, methyl eugenol, and methyl cinnamate	[49]
Eucalyptus essential oil	*Eucalyptus maideni*, *E. astrengens*, *E. cinerea*, *E. leucoxylon*	Leaves	1,8-cineol and α-pinene	[50]
Clove bud essential oil	*Eugenia caryophyllata*	Clove bud	Eugenol, eugenol acetate, and β-caryophyllene	[51]
Pine needle essential oil	*Cedrus deodara*	Pine needle	α-terpineol, linalool, limonene, anethole, caryophyllene, and eugenol	[52]
Lemongrass essential oil	*Cymbopogon citratus*	Leaves	Geranial, neral, and myrcene	[53]
Cinnamon bark essential oil	*Cinnamomum altissimum*	Bark	Linalool, methyl eugenol, limonene, α-terpineol, and terpinen-4-ol	[54]
Rosemary essential oil	*Rosmarinus officinalis*	Leaves	α-pinene, 1,8-cineole, and camphor	[43,55]
Black pepper essential oil	*Piper nigrum*	Fruit	α-pinene, sabinene, β-pinene, δ-3-carene, limonene, and β-caryophyllene	[43,56]
Peppermint essential oil	*Mentha piperita*	Leaves	Menthol and menthone	[5]

**Table 2:** Summary of recent studies on Essential Oil (EO)-containing packagings (EO-Aps).

Polysaccharide(s) Used	Essential Oil	Functional Activities of the Films	Food Product Analyzed	Reference
Okara soluble dietary fiber, pectin, sodium carboxymethyl cellulose	Thyme essential oil	Free radical scavenging, antibacterial activity (e.g., *Escherichia coli* and *Staphylococcus aureus*)	--	[14]
Chitosan	Clove essential oil	Antimicrobial activity (e.g., *Escherichia coli* and *Staphylococcus aureus*)	--	[11]
Chitosan	Cumin essential oil	Antimicrobial activity (e.g., *Listeria monocytogene*)	Beef loins	[66]
Chitosan	Garlic essential oil	Free radical scavenging, antibacterial activity (e.g., *Staphylococcus aureus*)	Sausages	[67]
Carboxymethyl cellulose, chitosan biguanidine hydrochloride	Frankincense essential oil	Antimicrobial activity (e.g., *Streptococcus pneumoniae, Bacillus subtilis, Escherichia coli*)	--	[16]
Chitosan, gum arabic	Cinnamon essential oil	Free radical scavenging	--	[68]
Shahri Balangu seed mucilage	Cumin essential oil	Antimicrobial activity (e.g., *Escherichia coli* and *Staphylococcus aureus*)	Beef slices	[69]
Fully deacetylated chitosan	Clove essential oil	Antimicrobial activity	White prawn shrimp	[70]
Basil-seed gum	Oregano essential oil	Free radical scavenging, antibacterial activity (e.g., *Escherichia coli*, *Staphylococcus aureus,Salmonella typhimurium, Pseudomonas aeruginosa, Bacillus cereus*)	--	[71]
Chitosan, pectin	Rosemary essential oil	Antimicrobial activity (e.g., *Bacillus subtilis, Staphylococcus aureus, Enterococcus aerogenes, Enterococcus faecalis, Escherichia coli*)	--	[72]
Alginate–apple puree edible film	Oregano oil, Cinnamon oil and Lemongrass oil	Antimicrobial activity (e.g., *Escherichia coli*)	--	[73]
Chitosan and cashew gum	*Lippia sidoides* oil	Larvicide (e.g., *Aedes aegypti*)	--	[74]
Cellulosic films	Cinnamon, Oregano and Lemongrass EO	Antifungal activity	--	[75]
Carboxymethyl cellulose	Garlic essential oil	--	Strawberries	[76]
Gelatin–Carboxymethyl Cellulose Film	Essential Oil of Bane	Antimicrobial activity (e.g., *Escherichia. coli, Salmonella enterica, Staphylococcus aureus, and Clostridium sporogenes*)	-- --	[77]
Carboxymethyl cellulose	Essential oils from Coriander, Rosemary and Nutmeg	--	--	[78]
Chitosan	Thyme essential oil	Antimicrobial activity (e.g., *Pectobacterium carotovorum*)	Bell Pepper	[79]
Sago Starch	Cinnamon essential oil	Antimicrobial activity (e.g., *Escherichia coli, Salmonella typhimurium, and Staphylococcus aureus*)	--	[80]
Chitosan	*Citrus limonia* essential oil	Antimicrobial activity (e.g., *Staphylococcus aureus*)	--	[81]
Dandelion polysaccharide	*Litsea cubeba* essential oil	Antimicrobial activity (e.g., *Staphylococcus aureus*)	--	[82]
Soybean polysaccharide	Ataria multiflora Boiss and Mentha pulegium essential oils	Free radical scavenging, antibacterial activity(e.g., *Staphylococcus aureus, Bacillus cereus, Escherichia coli, Pseudomonas aeruginosa*)	--	[83]
Hydroxypropyl methylcellulose	Oregano and bergamot essential oils	Antimicrobial activity (e.g., *Escherichia coli*)	Fresh ‘Formosa’ Plum	[84]
Sodium alginate	Garlic oil	Antimicrobial activity (e.g., *Escherichia coli*, *Salmonella typhimurium*, *Staphylococcus aureus* and *Bacillus cereus*)	--	[85]
Cassava starch	Cinnamon essential oil	Antimicrobial activity (e.g., *Escherichia coli*, *Salmonella typhimurium*, *Staphylococcus aureus*)	--	[86]
Sodium alginate	Thyme, lemongrass and sage essential oil	Antimicrobial activity (e.g., *Escherichia coli*)	--	[61]
Millet Starch	Clove Essential Oil	Antimicrobial activity (e.g., *Escherichia coli, Pseudomonas aeruginosa, Enterobacter* spp., *Bacillus cereus, Staphylococcus aureus*)	--	[87]
Chitosan	Clove essential oil	Antimicrobial activity (e.g., *Pseudomonas* spp. Enterobacteriaceae)	Pork Patties	[88]
Chitosan	Ginger Essential Oil	Free radical scavenging, Antimicrobial activity (e.g., *Bacillus cereus, Enterococcus faecalis, Listeria monocytogenes, Staphylococcus aureus, Escherichia coli, Pseudomonas aeruginosa, Salmonella enterica, Candida albicans*)		[89]
Chitosan	Limonene essential oil	Antimicrobial activity	Cucumber	[90]
Chitosan/CMC	*Mentha spicata* essential oil	Antibacterial activity (e.g., *Listeria monocytogenes*)	Strawberries	[91]
Alginate/CMC	*Ziziphora clinopodioides* essential oil	Antibacterial activity (e.g., *Listeria monocytogenes*)	Carp fillets	[92]
Starch	Clove leaf oil	Antioxidant activities and Antibacterial activity (e.g., *Listeria monocytogenes*)	Cheese	[93]
Banana flour (starch)	Garlic essential oil	Antioxidant activities and Antibacterial activity (e.g., *Aspergillus flavus*)	Peanuts (roasted)	[94]
